# *Caldicellulosiruptor saccharolyticus* transcriptomes reveal consequences of chemical pretreatment and genetic modification of lignocellulose

**DOI:** 10.1111/1751-7915.12494

**Published:** 2017-03-20

**Authors:** Sara E. Blumer‐Schuette, Jeffrey V. Zurawski, Jonathan M. Conway, Piyum Khatibi, Derrick L. Lewis, Quanzi Li, Vincent L. Chiang, Robert M. Kelly

**Affiliations:** ^1^ Department of Chemical and Biomolecular Engineering North Carolina State University Raleigh NC 27695 USA; ^2^ Department of Forestry and Environmental Resources North Carolina State University Raleigh NC 27695 USA; ^3^ Present address: Department of Biological Sciences Oakland University Rochester MI USA; ^4^ Present address: Novozymes Biologicals Durham NC USA; ^5^ Present address: State Key Laboratory of Tree Genetics and Breeding Chinese Academy of Forestry Beijing 100091 China

## Abstract

Recalcitrance of plant biomass is a major barrier for commercially feasible cellulosic biofuel production. Chemical and enzymatic assays have been developed to measure recalcitrance and carbohydrate composition; however, none of these assays can directly report which polysaccharides a candidate microbe will sense during growth on these substrates. Here, we propose using the transcriptomic response of the plant biomass‐deconstructing microbe, *Caldicellulosiruptor saccharolyticus*, as a direct measure of how suitable a sample of plant biomass may be for fermentation based on the bioavailability of polysaccharides. Key genes were identified using the global gene response of the microbe to model plant polysaccharides and various types of unpretreated, chemically pretreated and genetically modified plant biomass. While the majority of *C. saccharolyticus* genes responding were similar between plant biomasses; subtle differences were discernable, most importantly between chemically pretreated or genetically modified biomass that both exhibit similar levels of solubilization by the microbe. Furthermore, the results here present a new paradigm for assessing plant–microbe interactions that can be deployed as a biological assay to report on the complexity and recalcitrance of plant biomass.

## Introduction

The production of biofuels and bioproducts from renewable feedstocks has triggered efforts to develop plants and microorganisms that can be integrated into efficient bioprocessing schemes (Gronenberg *et al*., 2013; Loqué *et al*., 2015). When considering plant biomass feedstocks, one of the most significant challenges is to reduce recalcitrance of plant biomass either by thermo/chemical pretreatment (Sun *et al*., 2016) or through the use of synthetic biology and genetic tools to breed plants with reduced recalcitrance (Shih *et al*., 2016). On the microbial side, the focus is on metabolic engineering strategies that enable both plant cell wall deconstruction to fermentable sugars and subsequent conversion of these sugars to a biofuel at acceptable yields and titres (Liao *et al*., 2016). Given the potential scale of biofuel processes, even small improvements in carbohydrate accessibility and conversion can translate into significant economic benefits (Tao *et al*., 2011). Thus, it is important to have insightful methods to assess the subtle differences in plant cell wall characteristics, as these relate to biomass recalcitrance and potential toxicity arising from lignin moieties that are released during deconstruction.

Current efforts for improving the conversion of plant biomass to biofuels typically involve a thermal and/or chemical pretreatment step to reduce lignin content and to partially hydrolyse hemicellulose, components that shield cellulose microfibrils from microbial attack (Singh *et al*., 2015). Chemical pretreatments historically have relied on acidic (Tucker *et al*., 1998) or alkaline (Holtzapple *et al*., 1991) mechanisms for disrupting the bonds between lignin and polysaccharides. Alternatively, or in conjunction with chemical pretreatment, carbohydrate or lignin biosynthetic pathways in model biomass feedstocks have been genetically manipulated to produce transgenic plants with biochemically altered cell walls and consequently lowered recalcitrance to cellulose conversion (Van Acker *et al*., 2014). For example, genetically modified aspen (*Populus tremuloides*), in which a 4‐coumarate‐CoA ligase (*4CL*) (Chen and Dixon, 2007) lignin biosynthesis gene was downregulated, resulted in reduced lignin and increased cellulose levels, thereby simultaneously reducing recalcitrance and increasing substrate availability for saccharification. Genetic modifications to plant cell walls have improved conversion by microbes fermenting reduced lignin hybrid poplar (Li *et al*., 2003) and switchgrass (Van Acker *et al*., 2014). Furthermore, pairing less‐recalcitrant, genetically modified switchgrass with wild type *Clostridium thermocellum* (Fu *et al*., 2011) or an engineered and evolved strain of *C. thermocellum* (M1570) (Yee *et al*., 2014) increased ethanol production. Additionally, natural variation of plant cell wall composition in *Populus* sp. (Studer *et al*., 2011) has created feedstocks with improved conversion properties for consolidated bioprocessing (Dumitrache *et al*., 2016).

Whether chemical or genetic pretreatment is used or natural variants with favourable properties are considered, it is important to determine how the availability of the carbohydrate content of plant biomass to microbial attack has been modified and to what extent. Given the complexity of plant cell wall composition, which has defining biochemical and physical characteristics, it is difficult to determine subtle, but important, changes that can arise from chemical and genetic pretreatments. While a number of advanced analytical tools have been developed along these lines, they can only report on the sum total of chemical changes, rather than the differences that may elicit differential responses by microbes. When considering microbial conversion, however, no analytical method is capable of directly reporting the extent to which the particular feedstock has become more amenable to microbial attack. This might best be done using a microbial‐based assay.

Among the possible choices for such a microbial assay is *Caldicellulosiruptor saccharolyticu*s, a cellulolytic and xylanolytic bacterium, which is also capable of growth on a broad spectrum of carbohydrates (VanFossen *et al*., 2009) and acid‐pretreated (Blumer‐Schuette *et al*., 2010) and unpretreated biomass (Zurawski *et al*., 2015). Before‐and‐after analysis of the total carbohydrate content of feedstocks subjected to microbial treatment is capable of providing useful information on the suitability of a plant feedstock in supporting growth (Zurawski *et al*., 2015). Conversely, a more detailed view of compounds that the microbe senses and responds to (‘bioavailability’) could come from analysis of its transcriptome from growth on plant biomass feedstocks. Studies observing gene expression of either lignocellulosic fungi (Gaskell *et al*., 2014, 2016; Couturier *et al*., 2015) or lignocellulosic bacteria (Raman *et al*., 2009; VanFossen *et al*., 2011) grown on plant biomass feedstocks and sugars have so far focused on extracellular enzymes. Novel and useful insights into the plant biomass matrix in question could be obtained, based not only on the differential transcription of genes encoding glycoside hydrolases, but also including ATP‐binding cassette (ABC) sugar transporters and key metabolic pathway enzymes. Additionally, the transcriptome could reveal information concerning the impact of lignin modifications on the microbe, and whether this leads to a more easily deconstructed biomass. Here, we describe the use of *C. saccharolyticus* transcriptomes to probe for the bioavailable carbohydrate content of growth substrates and to evaluate recalcitrance of the substrate after chemical pretreatment or genetic modification of the plant biomass.

## Results and discussion

Before evaluating *C. saccharolyticus* transcriptomes to discern features related to biomass recalcitrance and deconstruction, it was first necessary to establish that this bacterium was capable of deconstructing the various forms of lignocellulose tested. *C. saccharolyticus* has previously been demonstrated to grow on dilute acid‐pretreated (DAP) switchgrass (*Panicum virgatum*), DAP *Populus* hybrid (*P. trichocarpa* x *deltoides*) (Blumer‐Schuette *et al*., 2010) and unpretreated switchgrass (Zurawski *et al*., 2015) to differing extents. Aside from previous biomasses tested, we also sought to establish that *C. saccharolyticus* was capable of growth on related unpretreated biomasses, including wild type (*P. trichocarpa*) and genetically modified *P. trichocarpa* samples. As such, we elected to focus on poplar and switchgrass as representative plant biomasses for this study.

### Solubilization of lignocellulose by *C. saccharolyticus*

**Figure 1:**
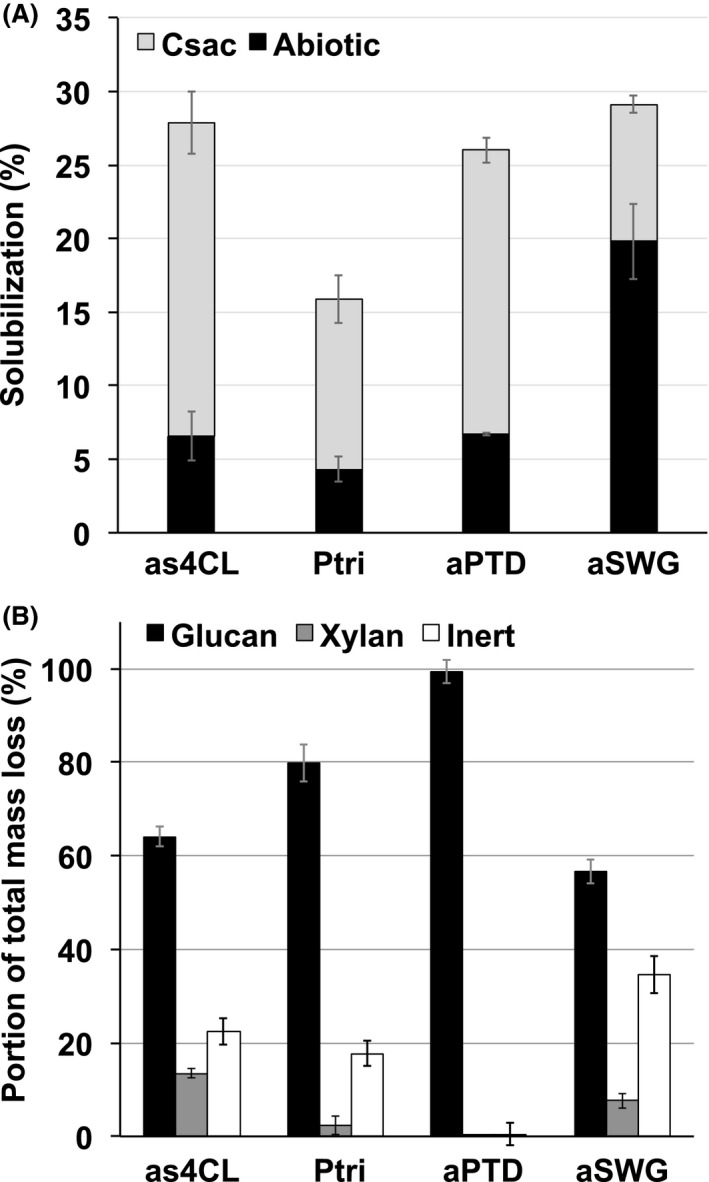
Solubilization of plant biomass by *Caldicellulosiruptor saccharolyticus*. A. Percentage of mass loss after 7 day incubation at 70°C. Each column represents percentage of mass loss due to biotic (*Csac*, grey) or thermal (abiotic, black) means for each biomass tested. B. Percentage of total mass loss attributed to glucans (black), xylan (grey) or inert material (white). Solubilization percentages are the means ± standard deviations (*n* = 3). as4CL, lignin‐reduced *Populus trichocarpa*; Ptri, unpretreated *P*. *trichocarpa*; aPTD, dilute acid‐pretreated *Populus trichocarpa* x *deltoides*; aSWG, dilute acid‐pretreated switchgrass.

Here, the solubilization of unpretreated *Populus* sp. (wild type *Populus trichocarpa*), genetically modified, lignin‐reduced *Populus* sp. (*4CL* antisense *P. trichocarpa*), DAP *Populus* and switchgrass was determined as a prelude to comparative transcriptomic analysis based on these plant biomasses. Mass loss attributed to *C. saccharolyticus* grown on lignin‐reduced or DAP *Populus* (21.3 ± 3.8% and 19.3 ± 1.0%, respectively, above abiotic controls) was higher than mass loss of wild‐type *Populus* or DAP switchgrass (11.5 ± 2.5% and 9.3 ± 3.1%, respectively, above abiotic controls, see Fig. 1A). It is interesting that the extent of biological solubilization for lignin‐reduced *Populus* either by chemical or by genetically modified means was comparable. The low level of biological solubilization during growth on DAP switchgrass can be partially attributed to the high level of abiotic solubilization (19.8 ± 2.6%), presumably due to water‐soluble oligosaccharides liberated by DAP.

Quantitative saccharification was then used to determine relative levels of glucans, xylan, arabinans and inert material before and after deconstruction by *C. saccharolyticus* for four types of plant biomass assayed (Table S1). Previous reports of biological solubilization by a related species, *C. bescii*, determined that the relative levels of glucans, xylans and lignin remained constant throughout subsequent passages (Kataeva *et al*., 2013). These data establish which types of biomass had reduced levels of recalcitrance and presumably more bioavailable carbohydrates that were in turn solubilized.

Prior to supporting growth of *C. saccharolyticus*, unpretreated biomasses (wild type and lignin‐reduced) contained more xylan on a per gram basis (Table S1) than did either of the DAP biomasses (*Populus* or switchgrass). Interestingly, when comparing genetically modified to wild‐type biomass, the overall percentage of biomass solubilized from lignin‐reduced *Populus* was higher (Fig. 1A) in addition to the relative proportion of both xylan and inert material liberated from lignin‐reduced *Populus* (Fig. 1B).

With respect to DAP biomass, the majority of solubilized carbohydrates from DAP *Populus* were glucans, while both glucans and xylan were solubilized during growth on DAP switchgrass (Fig. 1B), which shares similarity to lignin‐reduced *Populus*. Given that *C. saccharolyticus* was able to solubilize each biomass tested, we used unpretreated, DAP and genetically modified plant biomass as a basis for examining differences in transcriptomes during *C. saccharolyticus* active growth. Ultimately, we sought to determine whether our test case microbe, *C. saccharolyticus*, could detect differentiating features of recalcitrance and carbohydrate bioavailability as they impact potential biocatalyst performance.

### Complex polysaccharide transcriptomes

**Figure 2:**
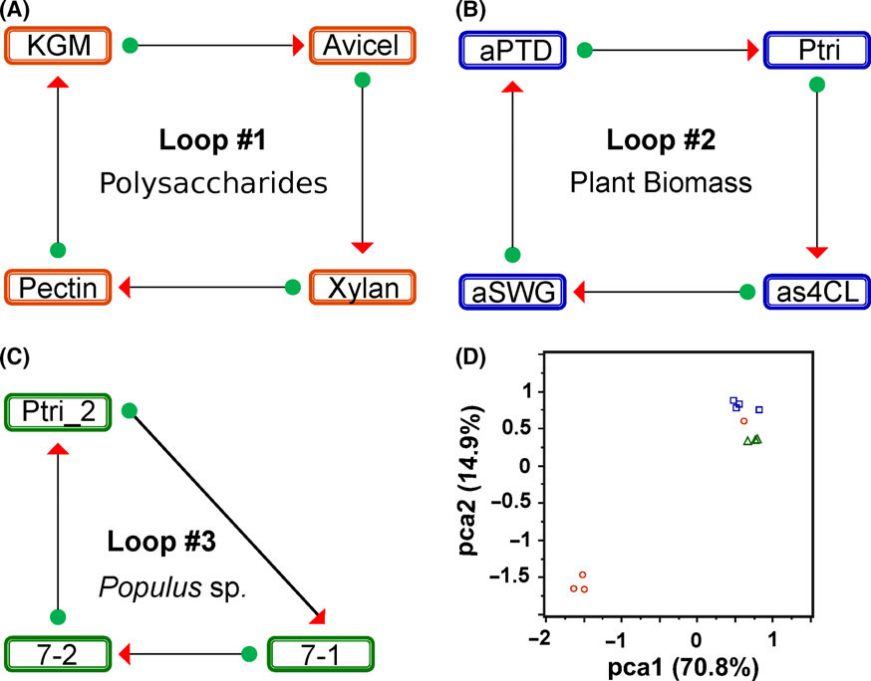
Transcriptional response experimental design and meta‐analysis. Experimental loop design is illustrated using arrows, which collectively represent a microarray slide hybridized with two treatments (Cy3 or Cy5 labelled). Arrows in panels A, B and C indicate the Cy3‐labelled (green circle) and Cy5‐labelled (red triangle) conditions respectively. Data from each loop were processed separately as described in experimental methods. A. Microarray Loop #1: KGM, konjac glucomannan; Avicel, crystalline cellulose; pectin; and xylan, birchwood xylan. B. Microarray Loop #2: aPTD, dilute acid‐pretreated *Populus* sp.; Ptri, unpretreated *Populus* sp.; aSWG, dilute acid‐pretreated switchgrass; as4CL, genetically modified *Populus* sp. C. Microarray Loop #3: Ptri_2, unpretreated Populus sp.; 7‐1 and 7‐2, genetically modified Populus sp., downregulated for cellulose levels. D. Two‐dimensional scatterplot of the first two principal components: blue squares, Loop #2; orange circles, Loop #1; green triangles, Loop #3. Principal components analysis (PCA) plot was compiled in JMP Genomics (SAS Institute, Cary, NC, USA).

To provide a basis for discerning subtle changes in plant cell wall composition as a result of natural variation, chemical pretreatment or genetic modification, the transcriptional response of *C. saccharolyticus* to purified forms of key plant polysaccharides was first determined (Loop #1, Fig. 2A). Crystalline cellulose was selected as one of the structural carbohydrates used in this bioassay as it is the major carbohydrate found in plant cell walls (Pauly and Keegstra, 2010). Hemicellulose components were also selected based on their presence in plant cell walls including xylan (Timell, 1967), glucomannan (Whitney *et al*., 1998) and pectin (Xiao and Anderson, 2013). This information was then used to probe *C. saccharolyticus* transcriptomes growing on plant biomass samples for differentiating features related to recalcitrance and carbohydrate availability for microbial utilization.

### Summary of the transcriptional response to complex polysaccharides

When comparing the global gene transcription patterns of *C. saccharolyticus* grown on model plant polysaccharides, crystalline cellulose triggered the largest transcriptional response, relative to the other carbohydrates tested. Approximately two‐third of the annotated open reading frames (ORFs) in the *C. saccharolyticus* genome responded for the contrasts between cellulose and the hemicelluloses: glucomannan, pectin or xylan. For example, 817 ORFs were upregulated and 900 downregulated during growth on crystalline cellulose when compared with xylan (Table S2). In addition, the global gene transcription profile of crystalline cellulose was also significantly different from other plant polysaccharides, as demonstrated by principal components analysis (Fig. 2D) and two‐way hierarchal clustering (Fig. S1). While crystalline cellulose induced the greatest physiological response among carbohydrates tested, *C. saccharolyticus* also responded selectively to hemicellulose with around 1/4 to 1/7 of genes from the *C. saccharolyticus* genome up‐ or downregulated in comparisons between glucomannan, pectin and xylan (Table S2).

**Figure 3:**
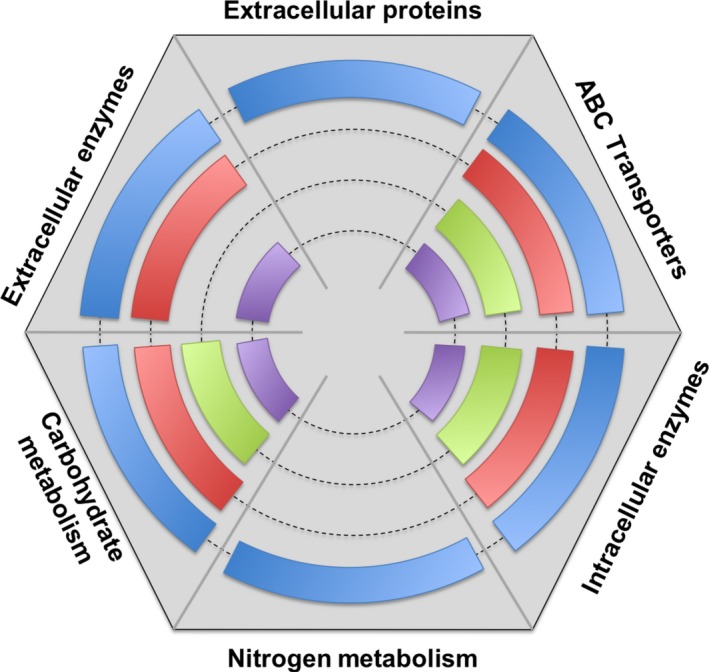
Representation of polysaccharide‐responsive genes from *C. saccharolyticus*. Each side of the hexagon corresponds to functional groupings of relevant genes. Colour blocks in inner rings indicate upregulation of genes within functional categories. Inner ring colours correspond to crystalline cellulose (outermost, blue); xylan (second ring, red); konjac glucomannan (third ring, green); and pectin (innermost ring, purple). For gene locus numbers in each functional category, refer to Table S6.

Insights into how *C. saccharolyticus* responds to purified plant biomass polysaccharides are useful in discerning its response to different plant biomasses. Major gene categories responding to the various purified plant polysaccharides are summarized in Fig. 3. This list includes extracellular enzymes that were highly upregulated on either cellulose or xylan (Table 1), ABC transporters (Table S3), intracellular enzymes (Table S4) and metabolic enzymes (Table S5) that were selectively upregulated on either cellulose, xylan, KGM or pectin.

**Table 1:** Extracellular Carbohydrate Active Enzymes from *Caldicellulosiruptor saccharolyticus*

						Fold change^a^					
Locus tag	Annotation/Activity	SigP	TM	AA	CAZy Family	Cellulose versus			Xylan versus		
						KGM	P	X	CC	KGM	P
Csac_0408	α‐Amylase	Y	N	514	GH13	–	–	–	4.3	–	–
Csac_0678	Endoglucanase/endo‐1,4‐β‐xylanase	Y	N	756	GH5, CBM28	12.8	11.4	8.8	–	–	–
Csac_0689	Pullulanase	Y	N	1136	CBM41, CBM48,GH13, CBM20	–	–	–	10.7	2.4	4.4
Csac_0696	Endo‐1,4‐β‐xylanase	Y	N	686	2× CBM22, GH10	–	–	–	148.6	35.7	31.3
Csac_0719	Acetyl xylan esterase	N	Y	299	CE4	–	–	–	–	–	–
Csac_1076	Endo‐1,4‐β‐glucanase (CelA)	Y	N	1751	GH9, 3× CBM3, GH48	2.2	7.4	3.3	–	–	2.2
Csac_1077	Endo‐1,4‐β‐glucanase/β‐1,4‐mannanase; endo‐1,4‐β‐xylanase (ManA)	Y	N	1303	GH5, 2× CBM3, GH44	60.9	89.3	35.1	–	–	2.6
Csac_1078	Endo‐1,4‐β‐xylanase/endo‐1,4‐β‐glucanase; β‐1,4‐glucomannanase (CelB)	Y	N	1039	GH10, CBM3, GH5	–	3.3	–	–	–	2.7
Csac_1079	Endo‐1,4‐β‐glucanase (CelC)	Y	N	1127	GH9, 3× CBM3	8.6	8.4	4.8	–	–	–
Csac_1081	Protein α‐mannosyltransferase	N	Y	565	GT39	–	2.9	–	–	–	–
Csac_1085	Endoglucanase/xyloglucanase	Y	N	931	GH74, CBM3	–	–	–	2.8	–	–
Csac_1560	Endo‐1,5‐α‐L‐arabinase	Y	N	488	GH43	–	–	–	20.8	2.7	5.8
Csac_2009	Acetyl xylan esterase	Y	N	258	CE4	–	–	–	–	–	–
Csac_2410	Endo‐1,4‐β‐xylanase (XynE)	Y	N	700	2 × CBM22, GH10				5.6	4.3	2.3
Csac_2411	α‐L‐Arabinofuranosidase (XynF)	Y	N	1347	GH43, CBM22, GH43, CBM6	2.3	2.5	1.9	–	–	–
Csac_2371	Acetyl xylan esterase	Y	N	322	CE4	–	–	–	–	–	–
Csac_2519	Carbohydrate binding protein	Y	N	628	CBM32	–	–	–	–	–	–
Csac_2528	Endoglucanase	Y	N	611	GH5	–	–	–	2.8	–	–
Csac_2722	Hypothetical protein/carbohydrate binding	Y	N	2593	GHnc	–	–	–	3.8	–	–

SigP, signal peptide; TM, transmembrane domain; AA, amino acid length; CC, crystalline cellulose; KGM, konjac glucomannan; P, pectin; X, xylan.

**a.** Fold change values are calculated from log_2_ ratios of crystalline cellulose or xylan versus other conditions listed in the columns below. Grey shading indicates statistical significance.

Overall, among all the candidate genetic markers of bioavailability, there are five upregulated, carbohydrate‐responsive loci that consist of CAZymes, their neighbouring ABC transporter and often metabolic enzymes, which serve as promising candidates to probe for the bioavailability of crystalline cellulose (Csac_0678, Csac_0679‐Csac_0681; GDL, Csac_1028‐Csac_1032), glucomannan (Csac_0294, Csac_0296, Csac_0297‐Csac_0301) and pectin (Csac_0354‐Csac_0356, Csac_0358‐Csac_0361). An ABC transporter that specifically responds to birchwood xylan (Csac_2692‐Csac_2694, 2696), but not oat spelt xylan (VanFossen *et al*., 2009), was also identified. In addition to enzymes and carbohydrate transporters, nitrogen metabolism loci were also upregulated in response to growth on crystalline cellulose (Fig. 3, Table S6). Moving forwards, the identified loci were used as probes to identify the bioavailability of polysaccharides in plant biomasses that were either chemically pretreated or genetically modified.

### Transcriptional response to plant biomass

As discussed above, the transcriptome of *C. saccharolyticus* was highly responsive to purified forms of plant biomass‐related polysaccharides as growth substrates, especially crystalline cellulose (see Fig. 3). The next step was to determine whether the bacterium's transcriptome reflected differences in recalcitrance and carbohydrate availability between DAP and unpretreated biomass feedstocks (Loop #2, Fig. 2B). In addition to the biomasses discussed above, an experimental loop was designed to look at genetically altered *Populus* species that were modified to reduce the amount of cellulose produced (Loop #3, Fig. 2C). The global transcriptional response of *C. saccharolyticus* to all biomass substrates tested clustered together with crystalline cellulose, but distinctly from xylan, pectin and glucomannan when using principal components analysis (Fig. 2D) or hierarchal clustering (Fig. S1). Furthermore, quantitative saccharification data also identified glucans as contributing the most to mass loss (Fig. 1B) during growth. Because the global transcriptional response of *C. saccharolyticus* during growth on crystalline cellulose resembles that of the response to plant biomass, crystalline cellulose appears to be overwhelmingly responded to by *C. saccharolyticus*, even in heterogeneous plant biomass. A summary of functional groups of genes upregulated in all plant biomass contrasts is found in Table 4.

While plant biomasses are a complex matrix of the same essential components, i.e. lignin, cellulose and hemicellulose, the hypothesis proposed here is that subtle compositional differences in the plant cell walls (naturally or through chemical pretreatment or genetic modification) should elicit detectable and differentiating transcriptional responses during degradation by *C. saccharolyticus*. Supporting this assumption, the number of genes whose expression levels changed by twofold or more was, as expected, significantly lower for the plant biomass contrasts, with at most 1.6% of total genes from the *C. saccharolyticus* genome responding (DAP versus lignin‐reduced *Populus*, Table 2). However, certain signatures were apparent that corresponded with chemical pretreatment or genetic modifications to which the various biomasses were subjected.

**Table 2:** Transcriptionally responsive ORFs (≥ twofold) for each biomass tested.^a^^,^ b

	*as4CL*	Ptri	aPTD	aSWG
*as4CL*	–	3	24	6
Ptri	21	–	28	3
aPTD	19	14	–	12
aSWG	30	2	26	–

as4CL, lignin‐reduced *Populus trichocarpa*; Ptri, wild type *P. trichocarpa*; aPTD, dilute acid‐pretreated *Populus trichocarpa* x *deltoides*; aSWG, dilute acid‐pretreated switchgrass.

**a.** log10 *p*‐value ≥ 5.59 used for statistical significance.

**b.** Fold changes are read as the treatment in columns versus the treatment in rows.

### Effect of dilute acid pretreatment (DAP)

As expected, we observed that DAP increased the biosolubilization of *Populus* by *C. saccharolyticus* from 11.5% (wild‐type) to 19.3% (DAP *Populus*) mass loss (Fig. 1B). Surprisingly, only 1.6% of genes from the *C. saccharolyticus* genome responded significantly to the differences between DAP and wild‐type *Populus* (Table 2). Given the chemical modifications to biomass during DAP, we expected that genes involved in hydrolysis or metabolism of simpler carbohydrates will be upregulated. The reduced recalcitrance of DAP *Populus* in comparison with wild type was confirmed with the upregulation of a monosaccharide ABC transporter (Csac_0240‐0242). In addition, we postulate that cellulose is now more bioavailable, as genes from the glutamate synthase locus and a predicted secreted protein (Csac_1052) were also upregulated, similar to the response observed for crystalline cellulose (Table S7).

Genes upregulated on unpretreated wild‐type *Populus* should reflect the recalcitrance of the tissue. Indeed, some markers of nutritional stress are upregulated during growth on wild type *Populus*, including annotated iron and iron sulfur proteins (Csac_0445, rubrerythrin; Csac_1990, rubredoxin), which have also been observed to be upregulated during stress by related clostridia (Hillmann *et al*., 2006; Venkataramanan *et al*., 2015). Aside from stress, stimulation of certain genes reflected the heterogeneity of unpretreated biomass. Genes corresponding to hemicellulose ABC transporters were upregulated (Csac_0297 and Csac_2696, respectively, see Tables S3 and S6), supporting quantitative saccharification data (Fig. 1B) that hemicellulose is bioavailable in wild type *Populus*. Genes encoding a putative GH20 family enzyme also responded; these were also upregulated on hemicelluloses in Loop #1 (Csac_2538, Tables S4 and S6). Additionally, xylose metabolism genes were upregulated, including a xylose isomerase (Csac_1154), consistent with the bacterium's hydrolysis and metabolism of hemicellulose components in the unpretreated biomass.

**Figure 4:**
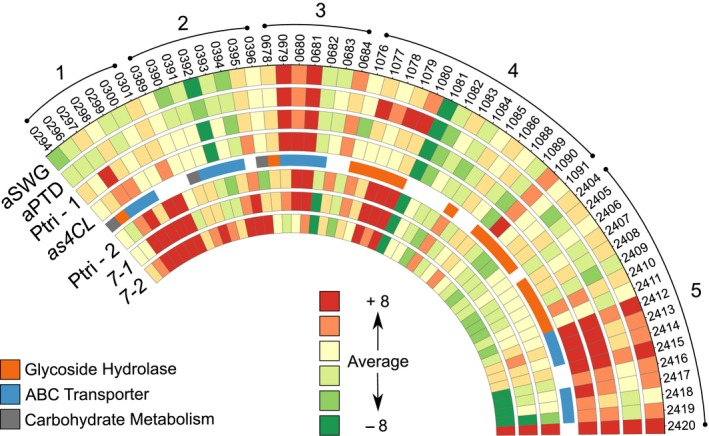
Heat plot comparing plant biomasses utilized by *C. saccharolyticus*. From outside to inside: aSWG, dilute acid‐pretreated switchgrass; aPTD, dilute acid‐pretreated *Populus* sp.; as4CL, lignin‐reduced genetically modified *Populus sp*.; Ptri ‐1 and Ptri‐2, *Populus sp*.; 7‐1 and 7‐2, cellulose‐reduced *Populus sp*. Sector 1, Csac_0292‐0294, Csac_0296‐Csac_0299; Sector 2, Csac_0389‐Csac_0402; Sector 3, Csac_0678‐Csac_0681; Sector 4, Csac_1076‐1080, Csac_1085, Csac_1091; Sector 5, Csac_2404‐Csac_2420. Gene loci included that are ABC transporters or glycoside hydrolases are indicated by blue and orange shading in the fifth ring. LSMeans values for genes represented here can be found in Table S7.

Aside from recalcitrance, the differential response of *C. saccharolyticus* to both DAP biomass types (switchgrass versus poplar) should reflect the previously observed differences in glucan and xylan composition (Fig. 1B) of the respective biomass. As expected, the largest fold changes with respect to DAP switchgrass centred on xylan hydrolysis and metabolism with genes upregulated for the transport (Sector 5, Fig. 4; Csac_2412, Csac_2419, Csac_2696) and metabolism of xylooligosaccharides (Csac_1154, Table S7). These results from the contrast between the two DAP samples confirm quantitative saccharification data (Table S1), demonstrating that more xylan is bioavailable in DAP switchgrass compared with DAP *Populus* and that the presence of both cellulose and xylan triggers a stronger response in the xylan degradation locus (XDL; Sector 5, Fig. 4), compared with cellulose or xylan alone (Table S7).

### Response to genetically modified Populus

We also sought to determine whether *C. saccharolyticus* would continue to prove useful as a microbial assay probing for differences in the bioavailability of polysaccharides between unpretreated biomass and genetically modified tissue. Biomasses with differing xylan, lignin and cellulose levels were assayed (Table S1), including *Populus* tissue with reduced levels of either lignin (*as4CL*) or cellulose (7‐1 and 7‐2). When comparing the transcriptional response of *C. saccharolyticus* to lignin‐reduced tissue versus wild type, we expected to observe genes responding similar to those observed in comparison of less‐recalcitrant DAP *Populus* with wild type.

Interestingly, while both lignin‐reduced and DAP *Populus* have reduced recalcitrance, the differential response of *C. saccharolyticus* grown on lignin‐reduced versus wild type *Populus* was in contrast to the comparison of DAP *Populus* versus wild type. Polysaccharides should be present in the lignin‐reduced *Populus* tissues that are absent from DAP *Populus* as a result of chemical hydrolysis during DAP. Strong upregulation of mannan metabolism genes (Sector 1, Fig. 4) and a xylan‐responsive ABC transporter gene locus (Csac_2692‐2694, 2696, Table S7) supports this assertion. Furthermore, higher levels of lignin‐reduced *Populus* biomass was solubilized by *C. saccharolyticus* (Fig. 1A), with a larger proportion of xylan solubilized in comparison with wild type (Fig. 1B).

Genetically modified biomass with lowered cellulose content (*Populus* sp. ‘7‐1’ and ‘7‐2’) was then used to determine whether *C. saccharolyticus* could distinguish between other genetic modifications related to polysaccharide abundance and recalcitrance. Similar to the earlier comparisons between genetically modified and unpretreated *Populus*, modest changes in gene expression were observed, with < 4% of total genes from the *C. saccharolyticus* genome responding to a reduction in the cellulose content of the biomass. The number of genes up‐ or downregulated also increased with the severity of cellulose reduction when compared with wild type (Table 3), and we expected to observe less cellulose‐responsive loci upregulated. The most striking differences were observed from the response of *C. saccharolyticus* to the most cellulose‐reduced tissue (7‐2, Table S1). Accordingly, some cellulose‐responsive genes were downregulated, including the genetic locus, Csac_0678‐Csac_0681 (Sector 3, Fig. 4), which includes cellulose‐responsive endoglucanase and ABC transporter. More so than upregulation of the GDL, this locus (Tables S6 and S7) appears to be a better indicator of the bioavailability of cellulose as detected by *C. saccharolyticus*.

**Table 3:** Transcriptionally responsive ORFs (≥ twofold) for each biomass tested.^a^^,^ b

	Ptri	7‐1	7‐2
Ptri	–	56	62
7‐1	10	–	17
7‐2	22	20	–

Ptri, wild type *Populus trichocarpa*; 7‐1, moderately cellulose‐reduced *P. trichocarpa*; 7‐2, severely cellulose‐reduced *P. trichocarpa*.

**a.** log10 *p*‐value ≥ 5.36 used for statistical significance.

**b.** Fold changes are read as the treatment in columns versus the treatment in rows.

Levels of xylan increased in the genetically modified tissue as cellulose was reduced (Table S1), and this appears to have also increased the recalcitrance of the cellulose‐reduced tissue (7‐1 and 7‐2). Most xylan‐responsive genes were repressed in response to growth on cellulose‐reduced tissue (7‐2), including enzymes and ABC transporters from the XDL (Sector 5, Fig. 4). Other polysaccharide‐responsive ABC transporters were upregulated instead, such as the predicted glucomannan transporter (Sector 1, Fig. 4: Csac_0297‐0301), an ABC transporter cluster with an unknown preferred substrate (Sector 2, Fig. 4: Csac_0391‐0394) and an α‐glucan ABC transporter (Table S3: Csac_0427‐0431).

Additional genetic loci that can serve as indicators of nutritional stress due to increased recalcitrance include α‐glucan transport/metabolism and chemotaxis. Turnover of stored α‐glucan reserves can be used as indicators of biomass recalcitrance, as the microbe will need to rely on previously stored carbohydrates to support growth. Comparing the most recalcitrant cellulose‐reduced biomass with wild type, many enzymes upregulated are involved in α‐glucan hydrolysis GH family 13, 15 and GT family 35 (Csac_0130, Csac_0203, Csac_0408, Csac_0426 and Csac_0429, see Table S7). In contrast, genes involved in the synthesis of glycogen were downregulated, supporting the assumption that the microbe is relying on energy stores for growth. Additionally, a predicted chemotaxis cluster was also upregulated in comparison with wild type (Csac_0811‐0812, 0814, 0816, Table S7). Previous reports identified the upregulation of homologous chemotaxis clusters in response to crystalline cellulose in *C. bescii* and *C. kronotskyensis*, but not *C. saccharolyticus* (Zurawski *et al*., 2015). In cases of extreme recalcitrance, as seen here, it appears that nutritional stress serves as an alternate signal for the genetic regulation of *C. saccharolyticus* chemotaxis genes (Table S7).

### Comparison between strategies to reduce biomass recalcitrance

Potential insights into the bioavailability of carbohydrates and recalcitrance were next examined for the transcriptional response of *C. saccharolyticus* to DAP and lignin‐reduced *Populus*, to ascertain any differences arising from chemical pretreatment and genetic modification. Because both tissues are less recalcitrant, we expected to observe differential regulation of polysaccharide‐responsive genes and not the previously identified stress loci. Surprisingly, there were more differences between DAP and lignin‐reduced *Populus*, which are genetically similar, than DAP switchgrass and lignin‐reduced *Populus*, which compares biomass across species. Genes found to be upregulated in response to lignin‐reduced *Populus* were indicative of the diversity of carbohydrates, primarily hemicellulose, left in unpretreated, genetically modified tissue. Gene loci previously identified as markers for the bioavailability of (gluco‐)mannan (Csac_0296‐Csac_0301; Sector 1, Fig. 4) and xylan (Csac_1154, Csac_2694‐Csac_2696; Table S7) were upregulated. Additional transporters previously tied to xylan transport (Zurawski *et al*., 2015) were also upregulated, including transporters from the XDL (Csac_2412; Csac_2417‐Csac_2419; Sector 5, Fig. 4). Besides ABC transporters, a resistance, nodulation and division (RND)‐type transporter (Csac_1474) was upregulated. This gene was also upregulated on both glucomannan and pectin (Table S7). Proteins containing this domain (MMPL, PF03176) often have no assigned function, although in a related clostridial species, *C. acetobutylicum*, a homologue of Csac_1474 was observed to be upregulated in response to fermentation products such as butanol (Schwarz *et al*., 2012), acetate and butyrate (Alsaker *et al*., 2010).

In contrast, the differential response to DAP *Populus* included upregulation of cellulose‐responsive enzymes in the GDL (Csac_1077‐Csac_1080) and both glutamate synthase clusters (Table 1). Other previously identified enzymes involved with cellulose hydrolysis and metabolism were equally induced by both DAP and lignin‐reduced *Populus*. Given the broad substrate preference of *C. saccharolyticus*, it appears that genetic modification of biomass has certain favourable attributes, as it reduces recalcitrance without deleting fermentable sugars, such as mannans and xylans. Additionally, *C. saccharolyticus* is able to balance its nitrogen needs better during growth on lignin‐reduced *Populus* biomass in comparison with DAP *Populus*, potentially reducing the levels of exogenous nitrogen that would need to be added through the culture medium.

## Conclusions

The main features of the transcriptional responses to chemically pretreated, genetically modified and unpretreated biomasses are summarized in Table 4. Connections between the transcriptional responses of *C. saccharolyticus* to various carbohydrate types (cellulose, xylan, glucomannan and pectin) allude to their bioavailability in plant biomass. With < 5% of the total genome from *C. saccharolyticu*s responding in any given comparison between plant biomass types, transcriptional data from comparisons between plant biomass‐related polysaccharides (Table S6) gave insights on how *C. saccharolyticus* responds to subtle compositional differences.

**Table 4:**
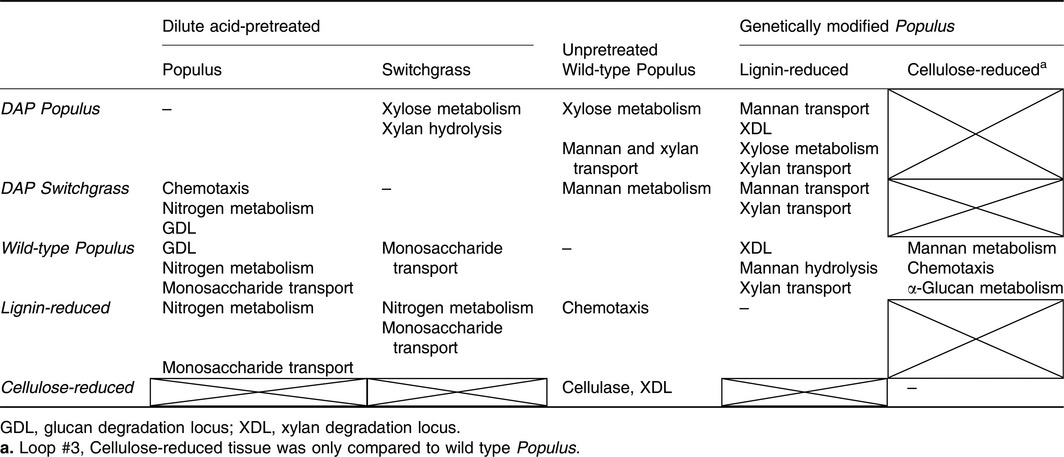
Characteristics of transcriptional response of *C. saccharolyticus* to selected plant biomasses

Chemical pretreatment improved solubilization of DAP *Populus* in comparison with wild type (Fig. 1) and also increased the bioavailability of cellulose in that comparison which was observed both in solubilization (Fig. 1B) and in transcriptional response (Table 4). DAP switchgrass provided more oligosaccharides for growth than wild type *Populus* (Fig. 1A), and genes involved in monosaccharide transport were accordingly upregulated (Table 4). However, it appears that these oligosaccharides from DAP switchgrass were readily soluble, resulting in lower biological solubilization of the insoluble biomass by *C. saccharolyticus* (Fig. 1A).

Comparing the response of *C. saccharolyticus* to DAP or genetically modified lignin‐reduced *Populus* (*as4CL*), more cellulose appears to be bioavailable in DAP *Populus* based on transcriptional response (Table 4). However, additional polysaccharides, such as mannan and xylan, that appear to be available in lignin‐reduced *Populus* are solubilized (Fig. 1B), and in addition, no nitrogen stress was apparent during growth (Table 4). Given that both biomasses were solubilized to similar extents (Fig. 1A), we propose that the genetically modified lignin‐reduced *Populus* tissue would be a superior biomass feedstock as it allows *C. saccharolyticus* to balance carbon and nitrogen needs. In comparison with genetically modified cellulose‐reduced tissue, the increased recalcitrance of cellulose‐reduced *Populus* (7‐1 and 7‐2) could be inferred by the downregulation of key hydrolytic loci: Csac_0678‐0680 and the XDL (Sectors 3 and 5, Fig. 4), and the upregulation of mannan metabolism genes and stress responses, chemotaxis and α‐glucan metabolism (Table 4).

In this proof‐of‐principle study, we elected to focus on two varieties of plant biomass (switchgrass and *Populus*) that had previously been demonstrated to support growth of *C. saccharolyticus*. Certainly, when considering alternate plant biomass feedstocks to support microbial growth, this system can be used to gain insights into the polysaccharides that are readily bioavailable for the microbe, in addition to highlighting other nutritional requirements for medium composition. Overall, we demonstrated the use of microbial transcriptome‐based methods for evaluating the conversion potential of unpretreated and genetically modified *Populus* varieties in comparison with DAP *Populus* and DAP switchgrass. Moving forwards, transcriptome‐based assays will become more comprehensive as additional data are generated and more is understood about the microbial biochemistry and metabolic processes of the indicator microorganism.

## Experimental procedures

### Microbial cultivation

*Caldicellulosiruptor saccharolyticus* (DSM 8903) was obtained as a freeze‐dried culture from the German Collection of Microorganisms and Cell Cultures [DSMZ (http://www.dsmz.de)]. After reanimation, *C. saccharolyticus* was maintained on DSMZ640 medium at 70°C with the following changes: Trypticase, FeCl_3_ x 6H_2_O, resazurin and cysteine‐HCl x H_2_O were omitted and 0.5% (w/v) Na_2_S x 9H_2_O was added as a reducing agent, and cellobiose was substituted with various carbon sources. Polysaccharides and biomass used include the following: crystalline cellulose (Avicel PH‐101, FMC); glucomannan (konjac); pectin (practical grade); birchwood xylan (Sigma); dilute acid‐pretreated switchgrass [*Panicum virgatum*, −20/+80 mesh fraction; pretreatment in a Sunds reactor at the National Renewable Energy Laboratory (NREL) (Raman *et al*., 2009)], dilute acid‐pretreated poplar [*Populus trichocarpa x deltoides*, provided by NREL (Jung *et al*., 2010)], *P. trichocarpa* and genetically modified *as4CL*, 7‐1 and 7‐2 *P. trichocarpa* (ground to 80 mesh in a Wiley mill). For the biomass microarray experiment, all polysaccharides and biomass were added at a concentration of 1 g l^−1^, noting that acid‐treated poplar and switchgrass were added at a wet weight. All cultures were subcultured four times on the applicable substrate in 50 ml batch cultures under N_2_ headspace in 125 ml serum bottles prior to inoculation for cell harvesting. Cell densities (cells per ml) were monitored using epifluorescence microscopy, as described before (VanFossen *et al*., 2009).

### Biomass solubilization and quantitative saccharification

Biomass substrates tested in microarray Loop #2 were also used to assess the solubilization ability of *C. saccharolyticus* as described by Zurawski *et al*. (2015). Solubilization is defined here as the amount of mass loss after 7 days corrected for the amount of mass loss by abiotic (thermal) factors. Mass loss was determined as the mass difference between the biomass used to prepare cultures (0.25 g) and insoluble biomass remaining after harvest. Quantitative saccharification of selected biomass before and after solubilization studies used a modified NREL protocol as previously described (Zurawski *et al*., 2015).

### RNA isolation and processing

Cultures (500 ml) in 1 l 45 mm diameter screw top bottles were inoculated with cells in exponential phase to a density of 1 × 10^6^ cells ml^−1^. Cells were harvested when the culture reached mid‐log phase, typically between 1–4 × 10^7^ cells ml^−1^, as determined by epifluorescence microscopy. In the case of biomass cultures, harvested culture was first filtered to remove insoluble plant material or polysaccharides through a coffee filter into chilled centrifuge bottles, then rapidly cooled in an ethanol‐dry ice bath, after which the cells were centrifuged at 4226 × *g* for 15 minutes and the cell pellet was stored at −80°C. RNA was extracted as described before (van de Werken *et al*., 2008) using TRIzol reagent (Invitrogen, Carlsbad, CA, USA) and columns from a RNeasy kit (Qiagen, Germantown, MD, USA). Biological repeats were used for all conditions, and total RNA was pooled prior to the reverse transcriptase reaction. For both four‐slide loop experiments, pooled total RNA was used for reverse transcription reaction with amino allyl‐labelled dUTP (Ambion, Carlsbad, CA, USA) as described in protocols devised by The Institute for Genomic Research (TIGR, ftp://ftp.jcvi.org/pub/data/PFGRC/MAIN/pdf_files/protocols/M009.pdf). Labelled cDNA was purified and conjugated to Cy3 or Cy5 reactive dyes (GE Healthcare, Marlborough, MA, USA) as described previously by TIGR for oligonucleotide microarrays (ftp://ftp.jcvi.org/pub/data/PFGRC/MAIN/pdf_files/protocols/M008.pdf). For hybridization to microarray slides, loop design was used, which requires labelling of cDNA from each experimental condition with Cy3 and Cy5 dyes, independently. Loop design allows for data from each experimental condition to be directly compared with each other, without the need for a single reference condition (Kerr and Churchill, 2001). Each microarray slide was then hybridized with Cy3‐ and Cy5‐labelled cDNA from two different experimental conditions, as seen in Fig. 2.

### Transcriptomic analysis

Oligonucleotide probes representing 2679 *C. saccharolyticus* ORFs were designed as described before (van de Werken *et al*., 2008). Probes were printed in‐house on UltraGAPS slides (Corning, Oneonta, NY, USA) using a Qarray^Mini^ arrayer (Genetix, New Milton, UK). Each oligonucleotide probe was spotted five times in a random pattern. Hybridized microarray slides were imaged with a GenePix 4000B microarray scanner (Molecular Devices, Sunnyvale, CA, USA), and signal intensity for each probe after excitation at 635 and 532 nm was calculated using genepix pro (6.0; Molecular Devices) before import into JMP Genomics 4.0 (SAS Institute, Cary, NC, USA). anova, loess or quantile normalization of intensity data was used prior to applying a mixed‐effects model statistical analysis (Wolfinger *et al*., 2001) as described previously (Pysz *et al*., 2004). Genes that were differentially transcribed twofold or more and also met a Bonferroni significance cut‐off equal to or below a −log10 (*p*‐value) of 5.36 (Loop #1) or 5.59 (Loops #2 and 3) are defined here as being either up‐ or downregulated. Further statistical analysis was conducted using JMP Genomics, including principal components analysis and hierarchal clustering (two‐way using the Ward method).

Data for both microarray loop design experiments were deposited with the Gene Expression Omnibus (GEO) database hosted at the National Center for Biotechnology Information (http://www.ncbi.nlm.nih.gov/geo/). The microarray platform designed for *C. saccharolyticus* is available as accession number GPL6681. The experimental series accession numbers are as follows: GSE90445 for Loops 1, 2 and 3. Accession numbers for experimental data are as follows: GSM2401354, GSM2401355, GSM2401356 and GSM2401357 for Loop #1; GSM2401358, GSM2401359, GSM2401360 and GSM240161 for Loop #2 and GSM240162, GSM240163 and GSM240164 for Loop #3.

## Conflict of Interest

None declared.

## Supporting information


**Fig. S1.** Hierarchical clustering of the global *C. saccharolyticus* transcriptional response to polysaccharide or plant biomass.

**Table S1.** Compositional analysis of biomass feedstocks used in this study.
**Table S2.** Significant changes in expression > 2‐fold for each complex polysaccharide tested^a,b^.
**Table S3.** LSMeans values for ABC transporter loci.
**Table S4.** Transcriptional response of intracellular CAZymes to polysaccharides.
**Table S5.** LSMeans Values of polysaccharide responsive genes.
**Table S6.** Major transcriptional responses of *C. saccharolyticus* to purified polysaccharides.
**Table S7.** LSMeans values of carbohydrate utilization proteins and biomass responsive genes.
