# Supplementary material for: Caldicellulosiruptor saccharolyticus transcriptomes reveal consequences of chemical pretreatment and genetic modification of lignocellulose
Source: Microb Biotechnol. 2017 Mar 20;10(6):1546–57. doi: 10.1111/1751-7915.12494 (PMC5658599; doi:10.1111/1751-7915.12494)
Supplement: Supplementary file 1 — Fig. S1. Hierarchical clustering of the global C. saccharolyticus transcriptional response to polysaccharide or plant biomass. [file MBT2-10-1546-s001.pdf]

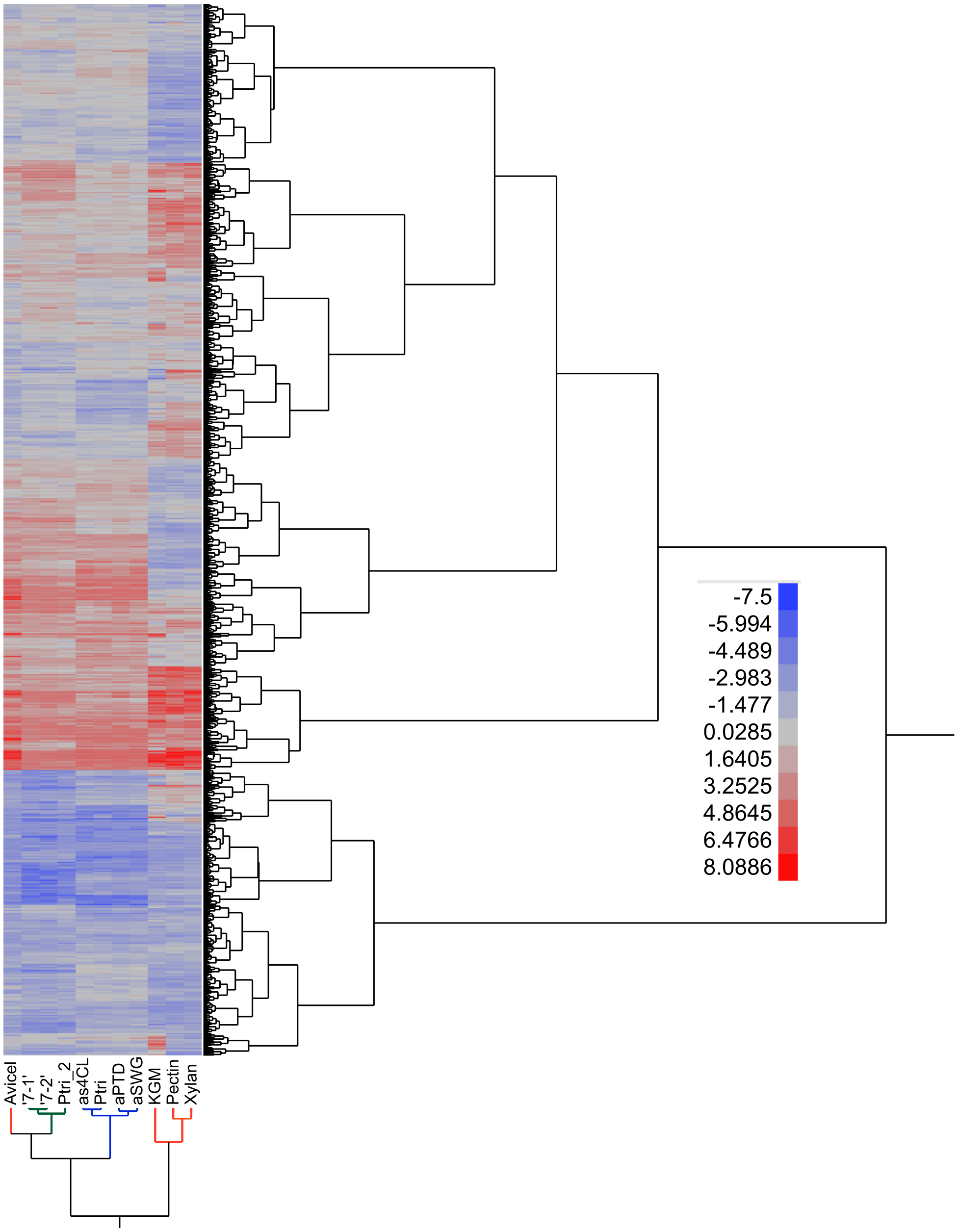

Figure S1. Hierarchical clustering of the global *C. saccharolyticus* transcriptional response to polysaccharide or plant biomass. Loop #1 (Avicel, crystalline cellulose; KGM, glucomannan; Pectin, technical grade and Xylan, birchwood), orange. Loop #2 (aPTD, acid pretreated *Populus* sp.; aSWG, acid pretreated switchgrass; Ptri, un-pretreated *Populus* sp.; *as4CL*, lignin-reduced *Populus* sp.), blue. Loop #3 (Ptri_2, un-pretreated *Populus* sp.; 7-1 and 7-2, cellulose-reduced *Populus* sp.), green. Two-way hierarchical clustering was compiled in JMP using Ward's method with LSMeans values for each ORF.
